# STARTING THE CONVERSATION: RESULTS FROM AN INTERPROFESSIONAL WORKSHOP ABOUT ADVANCE CARE PLANNING

**DOI:** 10.1093/geroni/igz038.3513

**Published:** 2019-11-08

**Authors:** Stacy L Barnes, Susan Breakwell, Jordan Cannon

**Affiliations:** 1 Marquette University, Milwaukee, Wisconsin, United States; 2 Medical College of Wisconsin, Milwaukee, Wisconsin, United States

## Abstract

Advance care planning is more than documenting end of life medical decisions; it should be the beginning of an important and ongoing conversation about personal values, goals, and preferences. Health profession students must be prepared to have these conversations and overcome any existing barriers to effective patient-provider communication. To this end, a multidisciplinary planning team from Marquette University and the Medical College of Wisconsin developed a 2-hour workshop directed at students in the health professions. It was designed to be highly interactive, including guided self-reflection, drawing, writing, videos, small group work, case presentations, and large group discussion. The workshop was scheduled to coincide with National Healthcare Decisions Day (April 16th). A total of 149 students participated, representing the disciplines of medicine, nursing, physician assistants, counseling psychology, speech pathology, and biomedical sciences. Feedback from both students and faculty was overwhelmingly positive, indicating interest and need for this type of program. A post-event questionnaire, which included a retrospective pre/post-test, assessed learners’ gains in knowledge and self-efficacy. Significant (< 0.001) gains were found on all measured items. Data from an electronic follow-up survey suggested the majority of participants took additional actions steps related to advance care planning in the month following the workshop. In conclusion, this is a low-cost, replicable workshop that aligns with current recommendations for advanced care planning (IOM, 2015), is well received by students and faculty in the health professions, and may serve as a springboard for increasing the number of advance care planning conversations.

